# Long non-coding RNA LOC285194 regulates vascular smooth muscle cell apoptosis in atherosclerosis

**DOI:** 10.1080/21655979.2019.1705054

**Published:** 2019-12-28

**Authors:** Qiushi Cheng, Min Zhang, Maoshen Zhang, Liang Ning, Dong Chen

**Affiliations:** a Department of Health Care, Qingdao Municipal Hospital (East), Qingdao, Shandong, China; b Department of General surgery, The affiliated Hospital of Qingdao University, Qingdao, Shandong, China

**Keywords:** Atherosclerosis, Apoptosis, Proliferation, Invasion, Migration, Long non-coding RNAs, Long non-coding RNA LOC285194

## Abstract

Long non-coding RNAs (lncRNAs) recently have been implicated in many biological processes and diseases. Atherosclerosis is a major risk factor for cardiovascular disease. However, the functional role of lncRNAs in atherosclerosis is largely unknown. Here we identified LOC285194 as a key regulator of cell proliferation and apoptosis during atherosclerosis. The expression of LOC285194 was dramatically down-regulated in a aortic atherosclerotic plaques of well-defined model of apolipoprotein-E knockout (ApoE^−/-^) mice. Moreover, we found that targeting LOC285194 results in neointimal hyperplasia *in vivo* in carotid artery injury model. We also showed that targeting LOC285194 promotes cell proliferation and inhibits apoptosis in vascular smooth muscle cells (VSMCs) *in vitro, and vice versa*. In addition, targeting LOC285194 promotes cell invasion and migration in vitro. Our studies identify LOC285194 as a novel regulator of cell proliferation and apoptosis and suggest that this lncRNA could serve as a therapeutic target to treat atherosclerosis and related cardiovascular disorders.

## Graphical Abstract

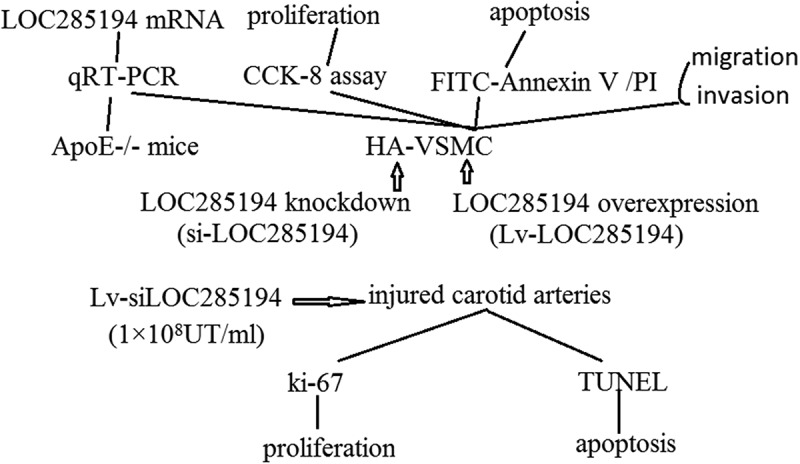

## Introduction

During progression of atherosclerosis, transformation of vascular smooth muscle cells (VSMC) from the quiescent contractile phenotype toward the proliferative migratory phenotype into the plaque area to form a fibrous cap is believed to be an essential step in the formation of stable plaques [1]. The two major phenotypes of VSMC include fully differentiated, contractile cells responsible for vasodilation and vasoconstriction, and migratory, proliferative cells that are activated during growth or injury [2].

During atherosclerosis development, VSMC respond to mediators such as platelet derived growth factor (PDGF)-BB, endothelin-1, thrombin, IFN-γ and IL-1 secreted by endothelial cells^3^ and leukocytes within the plaque [3], leading to migration from the vessel media to the intima where they secrete extracellular matrix components [4]. Advanced atherosclerotic plaques are characterized by the presence of fibrous cap, composed of VSMC and collagen, especially collagen type VIII, that surrounds and stabilizes the center core of the plaque, reducing the risk of plaque rupture and its consequences [5]. On the other hand, VSMC apoptosis leads to drastic vessel remodeling, with increased inflammation and coagulation, and thinning of the fibrous cap, making the plaque more prone to rupture [6].

Long non-coding RNAs (lncRNAs) are non-protein-coding transcripts longer than 200 nucleotides, which represent novel biomarkers for prognosis [7]. The expression of lncRNAs is frequently dysregulated in cancer [8] and participates in the proliferation, survival, migration, and invasion of cancer cells by modulating transcriptional, post-transcriptional, and epigenetic molecular events. Studies have revealed that targeting cancer-associated lncRNAs can impair cancer cell growth and metastasis [9]. Since lncRNAs are critical for the carcinogenic process, they could serve as novel therapeutic targets for cancer treatment [10].

Recently, lncRNA has also been reported to play a crucial role in cardiovascular diseases. Kumarswamy et al [11]. found that the expression of lncRNA uc022bqs.1 was increased in patients with heart failure and was associated with a higher risk of cardiovascular death. Chen et al. [12] reported that high expression of lncRNA NR_104181 and low expression of NR_027032 were possibly related to the risk of hypertension. LncRNA loc285,194 (LOC285194) was previously shown to be within a tumor suppressor unit in osteosarcoma and to suppress tumor cell growth [13]. It has recently found that LOC285194 that was weakly modulated in non end-stage patients was strongly down modulated in explanted failing hearts [14]. However, studies on the function of LOC285194 in VSMCs in atherosclerosis are still scarce. To do so, we made use of the apolipoprotein E knockout (ApoE^−/-^) mice. These mice are known to spontaneously develop atherosclerosis, even when fed a regular chow diet. The development and severity of atherosclerosis in these mice is increased further when they are fed a high fat diet, making the high fat diet-fed ApoE^−/-^ mice a useful model to study advanced atherosclerotic plaque development. Here, the initial aim of this study was to investigate the function of LOC285194 in the pathogenesis of atherosclerosis.

### Materials and methods

#### Cell culture

HA-VSMCs were obtained from American Type Culture Collection (Manassas, VA, USA) and cultured in F-12 K medium (American Type Culture Collection) containing 10% heat-inactivated fetal bovine serum (FBS; Gibco; Thermo Fisher Scientific, Inc., Waltham, MA, USA). Other nutrients added to the HA-VSMC culture were based on previously published literature [15]. Cells were incubated at 37°C with 5% CO_2_.

##### Lentivirus mediated siRNA gene knockdown LOC285194 or LOC285194 overexpression

Four RNA interference (RNAi) candidate target sequences were designed based on the human LOC285194 mRNA sequence and cloned into the pGCSIL-GFP vector (GeneChem, Shanghai, China). The RNAi sequence GAGTCGACACTCGCAAAGC was the most effective at suppressing LOC285194 mRNA in HA-VSMCs cells, and was used in subsequent experiments to knock down endogenous LOC285194. Nonsilencing (NS)-small interfering RNA (siRNA) (TACTCGGAACCTGTGAGCT) was also cloned into the pGCSIL-GFP vector and used as a control (GeneChem). A full-length sequence of LOC285194 gene was cloned into the pGCSIL-GFP vector as the manufacture’s instruction. The stable knockdown LOC285194 cell lines were generated by transduction a lentiviral mediated expression siRNA specific target of LOC285194 (si-LOC285194). The stable LOC285194 overexpressed cell lines were generated by transduction a lentiviral mediated expression LOC285194 (LOC285194). The virus transfected HA-VSMCs cell lines with 8 μg/ml polybrene. After 48 hours, the cells are harvested, and the transfection efficiency was tested by real-time-PCR.

##### RNA isolation and quantitative RT-PCR

Total RNA was extracted from the cultured cells using Trizol Reagent (Invitrogen, CA, USA). TaqMan miRNA Assay Probes (Applied Biosystems, Foster City, CA) were used according to the manufacturer’s instructions. Quantitative real-time PCR was performed using a TaqMan PCR kit on an Applied Biosystems 7500 Sequence Detection System (Applied Biosystems). All of the reactions were run in triplicate. After the reactions were complete, the cycle threshold (C_T_) data were determined using fixed threshold settings, and the mean C_T_ was determined from triplicate PCRs. A comparative C_T_ method was used to compare each condition to the control reactions. The selection of suitable reference genes as normalizers for relative quantification of loc285194 mRNA expression is essential to avoid erroneous expression results and to improve the comparability of gene expression data between different studies. The U6 snRNA (U6), RNU48, and Z30 were commonly used for expression normalization in the literature. The levels of loc285194 were normalized using RNU6B, as recommended in a variety of other studies. So we used U6 for expression normalization. U6 was used as an internal control, and the relative amount of miRNA normalized to U6 was calculated with the equation 2^−ΔΔCT^ in which ΔΔC_T_ = (C_T miR-19a_ − C_T U6_)_tumor_ − (C_T miR-19a_ − C_T U6_)_control_.

#### Animals

The apolipoprotein-E knockout (ApoE^−/-^) mice and wild mice were from our central laboratory. The injured carotid arteries model surgery with wild mice was performed following the procedures reported previously [16]. Briefly, Carotid artery surgery was performed under a dissecting microscope. The left carotid artery and its branches were exposed, and the left internal carotid artery (ICA) and the common carotid artery (CCA), distal to the bifurcation, were temporarily clamped. A transverse arteriotomy was made in the left external carotid artery (ECA), and a 33-G needle tip connected with a vascular catheter (Strategic Applications) was carefully inserted into the left ECA without any injury to the intima of the CCA. The CCA was washed three times with 100 μl of the base medium (saline with 0.2% BSA). Fourteen days later, local lentivirus-mediated gene transfer into injured carotid arteries was performed as described previously [16]. Briefly, 20 ul of recombinant lentivirus siRNA specific target of LOC285194 (1 × 10^8 ^UT/ml) and control siRNA (1 × 10^8^ UT/ml) were instilled into the common carotid artery and allowed to dwell for 30 minutes. Uninjured arteries were used as sham control. For cell proliferation and apoptosis, ki-67, immunofluorescence and TUNEL assays, respectively, were performed. Terminal deoxynucleotidyl transferase-mediated nick-end labeling (TUNEL) assays were performed on paraffin sections using the ApopTag® Plus In Situ Apoptosis Fluorescein Detection Kit according to the manufacturer’s procedure.

##### Cell proliferation assay

Cell proliferation was performed using CCK-8 assays. Briefly, 1 × 10^3^ cells were cultured in a 96-well plate at 37 °C. Plates were incubated at 37 °C for 2 h after each well was added with 10 *μ*l CCK-8 solution. Then, the spectrophotometric absorbance was measured at 570 nm for each sample. All the experiments were performed in triplicate and repeated 3 times, and the mean value was calculated.

##### Cell apoptosis assay

The transfected HA-VSMCs cells were harvested by trypsinization. Following the standard FITC-Annexin V and propidium iodide (PI) double staining procedure, the cells were analyzed by flow cytometry (BD Accuri C6; BD Biosciences, San Jose, CA, USA). The percentage of apoptotic cells was assessed (GuavaSoft 3.2). All of the samples were assayed in triplicate.

##### Cell migration and invasion assay

Cell migration and invasion potential was assessed by wound healing and transwell assays, respectively. For transwell assay, cells were trypsinized and 1 × 10^5^ cells in 100 *μ*l of serum-free RPMI-1640 medium were plated into the upper chamber. RPMI-1640 medium (500 *μ*l) supplemented with 20% FBS was added to the lower chamber. After culturing for 22 h, cells that had invaded the lower chamber were fixed with methanol and stained with 0.1% crystal violet. The number of invaded cells was observed by using an inverted microscope (magnification × 200) and calculated by counting five random views. For wound healing assay, cells were trypsinized and seeded in 6-well plates, and 12 h later an artificial wound was created by using a 200 μl pipette tip. The wound was observed after 24 h and imaged under a microscope. The fraction of cell coverage across the line was measured for the migration rate.

##### Statistical analysis

Data are presented as the mean ± standard error of the mean. Comparisons between two groups were performed using unpaired Student’s t-test and three or more groups were compared using one-way analysis of variance, followed by Dunnett’s test. P < 0.05 was considered to indicate a statistically significant difference.

## Results

### LOC285194 regulates cell proliferation and apoptosis

**Figure 1.**
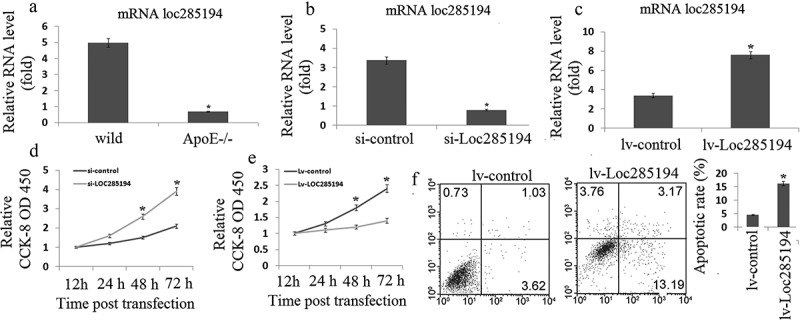
LOC285194 regulates proliferation and apoptosis in HA-VSMC cells. (a) LOC285194 transcript expression in atherosclerotic plaques of ApoE-/- mice and wild-type control mice (WT) was measured by qRT-PCR. It showed that the expression of LOC285194 was lower in atherosclerotic plaques of ApoE-/- mice compared to WT. (b) Si-RNAs were designed to knockdown LOC285194 in HA-VSMCs. Relative quantification of LOC285194 mRNA expression by qRT-PCR is shown as mean ± SD of three independent experiments. (c) The expression of LOC285194 was quantified by qRT-PCR in HA-VSMC cells transfected into Lv- LOC285194 or Lv-control. (d) The proliferation and viability of HA-VSMC cells were measured using the Cell Counting Kit-8 (CCK-8) colorimetric assay after LOC285194 knockdown. (e) The proliferation and viability of HA-VSMC cells were measured using the Cell Counting Kit-8 (CCK-8) colorimetric assay after LOC285194 overexpression. (f) The cell apoptosis of HA-VSMC cells were measured using the Annexin-V conjugated FACS analysis after LOC285194 overexpression. * P < 0.05 relative to control.

Given the vital role of p53 in the pathogenesis of atherosclerosis and the recent report that p53 regulates the expression of LOC285194 [17], we hypothesized that LOC285194 is also involved in the development of atherosclerosis. We first examined the expression of LOC285194 in aortic atherosclerotic plaques of ApoE-/- mice fed a high-fat diet, a widely used animal model of atherosclerosis. Indeed, we found that the expression of LOC285194 was substantially lower in atherosclerotic plaques of ApoE-/- mice (0.7 ± 0.2) when compared with that of wild type control mice(4.5 ± 1.3), suggesting that LOC285194 may play a role in atherosclerosis (Figure 1(a)).

Next, we investigated the function of LOC285194 in cell proliferation and apoptosis. We used the human vascular smooth muscle cell line HA-VSMC, which has been widely used to study atherosclerosis *in vitro*. We designed small interfering RNA (siRNA) to inhibit human LOC285194 (si-LOC285194) expression; We transducted a lentiviral mediated expression LOC285194 (LOC285194) to increase LOC285194 expression. The efficiency of siRNA or LOC285194 transfection and the inhibition or increase of endogenous LOC285194 were tested and confirmed by qRT-PCR (Figure 1(b,c)). Inhibition of LOC285194 substantially increased cell proliferation in HA-VSMC cells (OD450:2.8~4.3 vs OD450:1.4~1.6) (Figure 1(d)). LOC285194 overexpression substantially decreased cell proliferation (OD450:1.1~1.4 vs OD450:1.7~2.5) (Figure 1(e)) and increased apoptosis (14.6 ± 1.8 vs 3.7 ± 0.8)in HA-VSMC cells (Figure 1(f)). Together, these data indicate that LOC285194 suppresses cell proliferation and induces apoptosis.

### LOC285194 regulates cell invasion and migration

**Figure 2.**
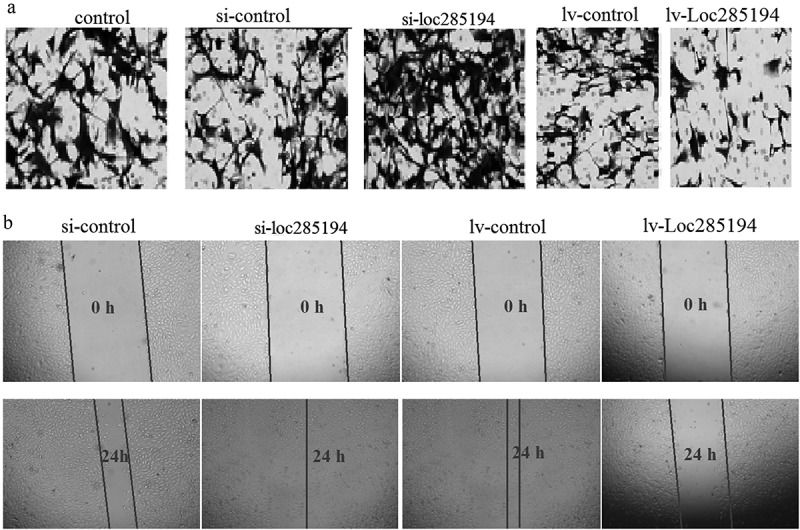
LOC285194 regulates cell invasion and migration in HA-VSMC cells in vitro. (a) Knockdown of LOC285194 by LOC285194 siRNA increased invasion in HA-VSMC cells in vitro by transwell assays; LOC285194 overexpression by Lv- LOC285194 transfection decreased invasion in HA-VSMC cells in vitro by transwell assays. Representative images at 24 h of the transwell assay are shown (×200). (b) Knockdown of LOC285194 by LOC285194 siRNA increased migration in HA-VSMC cells in vitro by wound healing assays; LOC285194 overexpression by Lv- LOC285194 transfection decreased migration in HA-VSMC cells in vitro by wound healing assays. Representative images at 0 h and 24 h of the wound-healing assay are shown (×4).

The invasion was assessed by Transwell invasion assay, and the number of invasive HA-VSMC cells was significantly increased after si-LOC285194 transfection (63.7 ± 4.6 vs 18 ± 5.6) (Figure 2(a)), and significantly decreased after Lv–LOC285194 transfection (28.6 ± 7.5 vs 6.3 ± 1.2) (Figure 2(a)). Furthermore, the cell migration was then assessed by wound healing assay, and the wound closure was significantly increased in HA-VSMC cells transfected with si-LOC285194 (93%±6.5% vs 78%±7.4%) (Figure 2(b)). LOC285194 overexpression substantially decreased migration in HA-VSMC cells (87%±11% vs 26.3%±4.6%) (Figure 2(b)).

### LOC285194 inhibits neointima formation in carotid arteries

**Figure 3.**
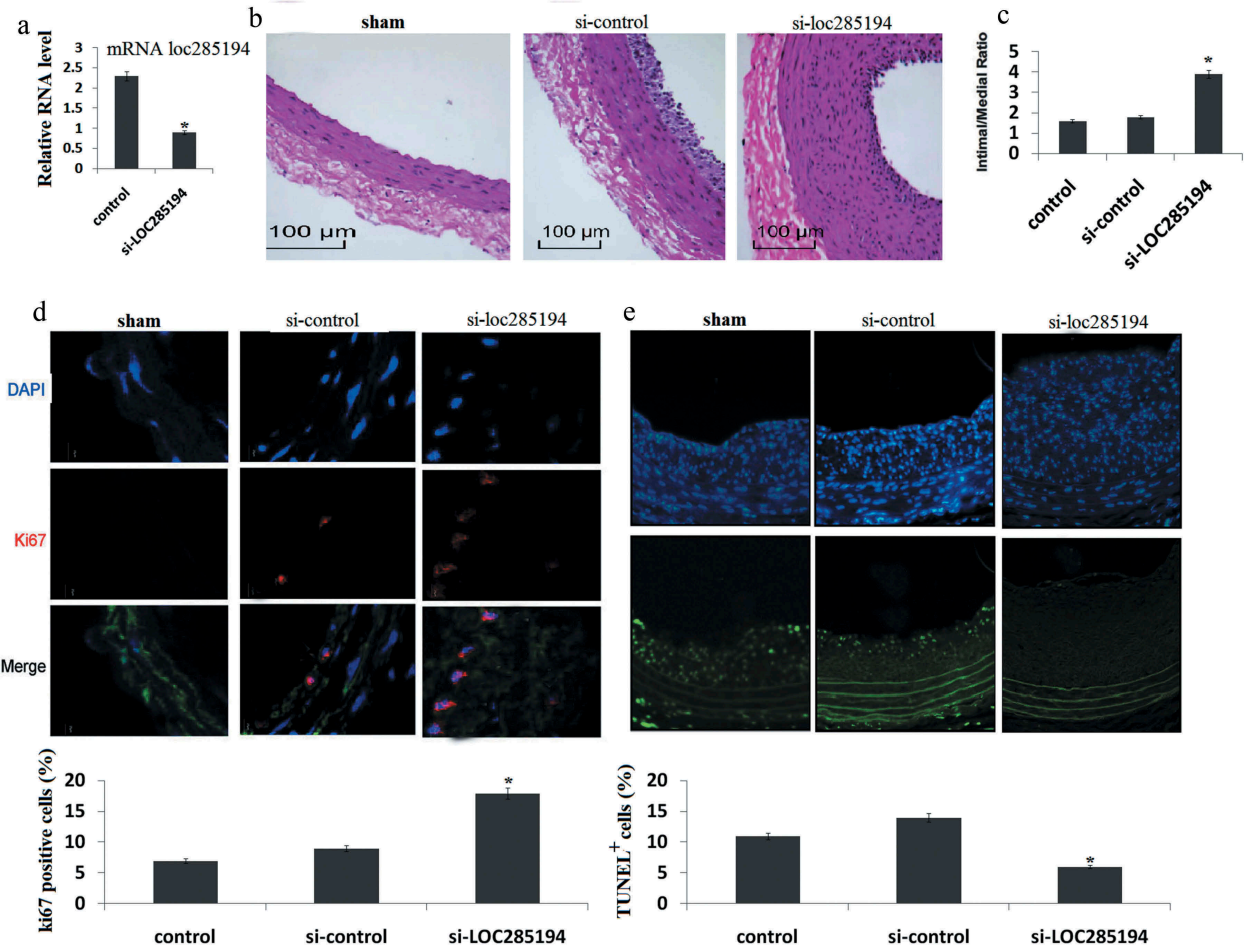
Inhibition of LOC285194 results in increased neointima formation. LOC285194 transcript expression in injured area of mouse carotid arteries measured by qRT-PCR. (a)LOC285194 mRNA expression was significantly higher in injured area of mouse carotid arteries compared to the control. (b) Lentivirus vectors for LOC285194 knockdown (si- LOC285194), or control si-RNA were in-site injected into the injured area of mouse carotid arteries. Sham operation serves as controls. Carotid arteries were harvested 30 days later after feeding mice with high-fat diet. H&E staining was performed to show the thickness of neointima. (c) Quantification of the intima-media thickness of sham, control si-RNA and si- LOC285194 treated samples. (d) Representative immunofluorescence images of Ki67 in mouse carotid arteries. DAPI staining marks cell nuclei. Quantification of the Ki67 positive signals of sham, control siRNA and si-LOC285194 treated samples. (e) Representative immunofluorescence images of TUNEL staining in mouse carotid arteries. DAPI staining marks cell nuclei. Quantification of the TUNEL positive signals of sham, control si-RNA and si- LOC285194 treated samples. All values are the average of at least 3 biological replicates and data shown are the mean±SD. * P < 0.05 relative to control.

Next, we investigated the involvement of LOC285194 in the formation of neointima *in vivo*, using the classic murine carotid artery injury model. Recombinant lentivirus vector expressing si- LOC285194 or control siRNA was injected into the injured area of mouse carotid arteries. These mice were then fed with high fat diet for one month, and neointima formation was examined. We verified the reduction of LOC285194 expression in local-injury carotid tissues after si-LOC285194 injection (Figure 3(a)). Knockdown of LOC285194 resulted in dramatic neointimal hypertplasia when compared with controls (Figure 3(b)). Quantification of intima-media thickness confirmed a significant increase after si-LOC285194 injection (Figure 3(c)). We asked whether inhibition of LOC285194 affected cell proliferation and apoptosis *in vivo*. We performed immunostaining on vessel sections to detect the proliferation marker Ki67. The fraction of Ki67^+^ cells increased in si-LOC285194 treated vessels (Figure 3(d)). si-LOC285194-treated vessels also showed decreased apoptosis, as assessed using the terminal deoxynucleotidyl transferase-mediated nick-end labeling (TUNEL) assay (Figure 3(e)).

## Discussion

The stability of the atherosclerotic plaque depends upon the thickness of the fibrous cap and the degree of cap inflammation. Plaque rupture is increased by cap thinning promoted by death of VSMCs and breakdown of collagen and extracellular matrix (ECM), which may subsequently lead to myocardial infarction or stroke [18]. However, plaque rupture is frequently sub-clinical, as VSMCs repair the rupture and reorganize the associated thrombus. Indeed, complicated plaques frequently show evidence of multiple ruptures and repair, ultimately resulting in luminal narrowing. Successful plaque repair requires VSMCs to proliferate and synthesize matrix, both properties that are altered by death and cellular senescence. Indeed, the balance of cell proliferation and migration vs. cell death and cell senescence determines the population of VSMCs within the atherosclerotic plaque. The role and regulation of these processes is crucial to both atherogenesis and plaque stability.

Increased cell proliferation is observed during early atherogenesis and upon vascular injury, and aged VSMCs from rodents also show increased proliferation [19,20] compared with cells from younger animals. In contrast, human VSMCs derived from both aged vessels and advanced atherosclerotic plaques undergo reduced proliferation and prolonged population doubling times [21]. This observation corresponds to *in vitro* findings where plaque VSMCs in culture show decreased percentages in S-phase and increased percentages in G_1_, consistent with a G_1_ growth arrest [22].

Loc285194, also called LSAMP antisense RNA 3, is an lncRNA consisting of 4 exons with >2 kbs in length (Gene ID: 285194) and is located at osteo3q13.31. As the osteo3q13.31 locus harbors frequent focal copy number alterations (CNAs) and loss of heterozygosity in primary osteosarcoma samples, it implies that loc285194 may function as a potential tumor suppressor. Furthermore, the tumor suppression function of loc285194 was also suggested by knockdown experiments, which showed an increased cell proliferation [13].

VSMC proliferation in atherosclerosis appears to be predominantly reparative, even in atherogenesis, and not the primary driver of plaque formation. The role of VSMC migration *per se* in atherosclerosis is still unclear, including adventitial progenitor populations. In contrast, VSMC cell death and cell senescence promote both atherogenesis and multiple features of plaque instability. Whether loc285194 mediates proliferation and apoptosis of vascular smooth muscle cells (VSMCs) in atherogenesis and plaque stability is unknown. In our study, we found the expression of LOC285194 was substantially lower in atherosclerotic plaques of ApoE-/- mice, suggesting that LOC285194 may play a role in atherosclerosis. We also found that overexpression of loc285194 lead to decreased proliferation in HA-VSMC cells, and vice versa. The presence of apoptosis in atherosclerotic plaques has been confirmed by a number of studies [23]. In our study, we found that targeting loc285194 with si-LOC285194 transfection reduced significant cell apoptosis in HA-VSMC cells *in vitro*.

The presence of a large number of intimal VSMCs, for example forming a fibrous cap, has been taken as evidence that VSMC migration from the media plays an important role in atherogenesis. However, VSMC migration is a difficult process to quantify in human atherosclerosis. It has shown that human VSMCs can migrate to a variety of stimuli in culture, but the contribution of VSMC migration to the mature atherosclerotic plaque is unclear. It is not clear whether migration occurs independently or is dependent upon cell proliferation. In our study, we found that targeting loc285194 promoted invasion and migration in cultured HA-VSMC cells *in vitro*, and vice versa.

Most of the research data have demonstrated that vascular remodeling is mainly caused by increased VSMC proliferation and decreased VSMC apoptosis after vessel injury. VSMC apoptosis in atherosclerosis has profound consequences, promoting multiple features of vulnerable plaques [24] such as a thin fibrous cap, enlarged necrotic core, and macrophage infiltration into the cap. We showed that targeting loc285194 increases cell proliferation and reduces apoptosis *in vivo*. Knockdown of endogenerous loc285194 accelerated neointima formation in injured carotid arteries, supporting our *in vitro* data. This finding is significant because it implicates non-coding RNAs in cardiovascular diseases such as atherosclerosis, and suggests that modulation of the activity of non-coding RNAs such as loc285194 may be a novel therapeutic approach to treat human cardiovascular disease. However, the limitations remain in the systemic applications of RNAi-based therapeutics. These hurdles should be addressed and resolved for the rational design of delivery vehicles for the targeted drug delivery to VSMCs cells in the future.

### Conclusion

Our studies identify LOC285194 as a novel regulator of cell proliferation and apoptosis and suggest that this lncRNA could serve as a therapeutic target to treat atherosclerosis and related cardiovascular disorders.

## Highlight

- LOC285194 inhibits proliferation and promotes apoptosis in HA-VSMCs cells *in vitro*.
- Targeting LOC285194 promotes proliferation and reduces apoptosis in HA-VSMCs cells *in vitro*.
- LOC285194 inhibits invasion and migration in HA-VSMCs cells *in vitro*, and vice versa.
- LOC285194 was downexpressed in atherosclerotic plaques of ApoE-/- mice
- Targeting LOC285194 results in neointimal hyperplasia *in vivo* in a carotid artery injury model.
